# Michigan State Board of Examiners in Dentistry

**Published:** 1896-06

**Authors:** 


### Michigan State Board of Examiners in Dentistry.

OFFICERS: A. T. Metcalf, D.D.S., President, Battle Creek; H. K. Lathrop, Jr., D.D.S., Treasurer, Detroit; Geo. H. Mosher, D.D.S., Secretary, Jackson.

### THE MICHIGAN DENTAL LAW.

The people of the State of Michigan enact as follows:

SECTION 1. That it shall hereafter be unlawful for any person to practice dentistry in this State unless such person has received a certificate of qualification from the Board of Examiners provided by this act; *Provided*, That the provisions of this act shall in no way apply to or affect any person who is now located and lawfully in actual practice in this State; *Provided further*, That a certificate shall be issued by said Board of Examiners to any one who has received a diploma from the faculty of a reputable dental college, duly incorporated under the laws of this or some other State of the United States, with a course of instruction and practice fully equal or equivalent to that of the college of dental surgery of the University of Michigan.

SEC. 2. Said Board of Examiners shall be appointed by the Governor of this State, and shall consist of three practical dentists who shall be regular graduates of a reputable dental college, duly incorporated under the laws of this State or some other State of the United States, or otherwise possess the necessary qualifications contemplated by this act.

SEC. 3. Each member of this Board of Examiners shall serve for a term of three years, and until his successor is duly appointed and qualified; except in the case of the first Board, the members thereof shall serve respectively one, two and three years as specified in the appointment of the Governor.

SEC. 4. The Board of Examiners shall be organized as follows: The member having but one year to serve shall be president of the Board; the one having two years, shall be treasurer, and the one having three years, shall be secretary. The treasurer shall make and file with the Secretary of State a good and sufficient bond to the people of the State of Michigan, in the penal sum of one thousand dollars, conditioned that he will well and truly pay over all moneys received by him as such treasurer, in compliance with the provisions of this act and otherwise faithfully discharge the duties of his office.

SEC. 5. The Board of Examiners shall meet at least once in each year, for the purpose of examining applicants, after having given, personally or by mail, thirty days' written or printed notice to each practicing dentist in the State who had filed his name and address with the secretary of said Board. The said Board is authorized to incur all necessary expenses in the prompt and efficient discharge of its duties, and pay the same with any moneys in the hands of its treasurer.

SEC. 6. Each member of said Board shall qualify by taking the oath of office prescribed by the Constitution of this State, and filing the same with the Secretary of State, before entering upon the duties of his office. Should a vacancy occur in said Board the Governor of this State shall fill the same by appointment.

SEC. 7. Any member of said Board of Examiners may, when the Board is not in session, examine applicants, and in case any applicant is found competent, grant a license to him to practice dentistry in this State until the next meeting of said Board, and no longer. Each applicant so examined shall pay the sum of three dollars. *Provided*, That no member of the said Board shall grant a license to any one who has been rejected on examination by the Board.

SEC. 8. Should any member of said Board be unable to attend at the meeting of the Board for the examination of applicants, he may appoint in writing a substitute, who shall have the same power on the examination that the member appointing him would have, if present. *Provided*, Such substitute be a person eligible to be a member of said Board within the provisions of this act. *And provided further*, That the appointment of such substitute be by and with the written consent of the other members of the Board.

SEC. 9. Each applicant for examination by the Board shall pay into the treasury of the Board the sum of ten dollars, which shall constitute a fund to defray the expenses of the Board; and each member of the Board shall receive therefrom the sum of three dollars per day for services rendered as such examiner. The Board shall keep a list of the names of all persons to whom licenses have been granted under the provisions of this act, and also of all persons practicing dentistry in this State in a book provided for that purpose, with the names arranged in alphabetical order.

SEC. 10. Any sum in excess of one hundred dollars which, under the provisions of this act, may accumulate in the treasury of said Board, shall be paid by the treasurer thereof into the treasury of this State.

SEC. 11. Each person now engaged in the practice of dentistry in this State shall, within ninety days after this act take effect, send an affidavit to the secretary of the Board setting forth his name, place of business, post-office address, the length of time he has been engaged in practice in this State, and if a graduate of a dental college, state the name of the same, and also pay to the treasurer of said Board the sum of twenty-five cents, and on failure to comply with said provisions of this section he shall be required to appear and be examined by said Board.

(NOTE.—The above Section 11, was in no way altered or changed by the amendments of 1891, and applied only to persons in practice at the time of the passage of the original act in 1883.)

SEC. 12. Any person who practices dentistry in this State, in violation of the provisions of this act, shall be deemed guilty of a misdemeanor, and upon conviction thereof shall be fined not less than twenty-five dollars, nor more than one hundred dollars, or sentenced to imprisonment in the county jail for a period not exceeding ninety days, or both, such fine and imprisonment is in the discretion of the court. *Provided*, That nothing in this act shall be construed so as to interfere with physicians and surgeons in their practice as such.

SEC. 13. For the purposes of instruction students may be employed to assist in dental offices, and in the College of Dental Surgery of the University of Michigan, under the immediate observation and advice of the legal proprietors and professors thereof, but no person not legally qualified and registered under this act shall assume the charge and management of any dental office, or the responsibility of deciding upon or the doing of dentistry at any private residence or elsewhere.

SEC. 14. All persons not now registered, who desire to practice dentistry in this State, shall apply to the secretary of the Board for registration. Each person seeking registration by virtue of a diploma shall send an affidavit to the secretary of the Board, setting forth his name, place of business, post-office address, the date of his graduation, and the name of the dental school from which graduated, and a registration fee of three dollars.

All applicants found qualified under this act shall be properly and promptly registered by the secretary of the Board.

SEC. 15. The secretary of said Board shall, on the first day of June of each year hereafter, file an annual report with the Secretary of State, showing the number of applicants for examination, the number who passed said examination and received a license to practice, the amount of the fees received from the applicants from such licenses granted, and the amount received by each member for his expenses and services.

Approved May 10, 1893.

